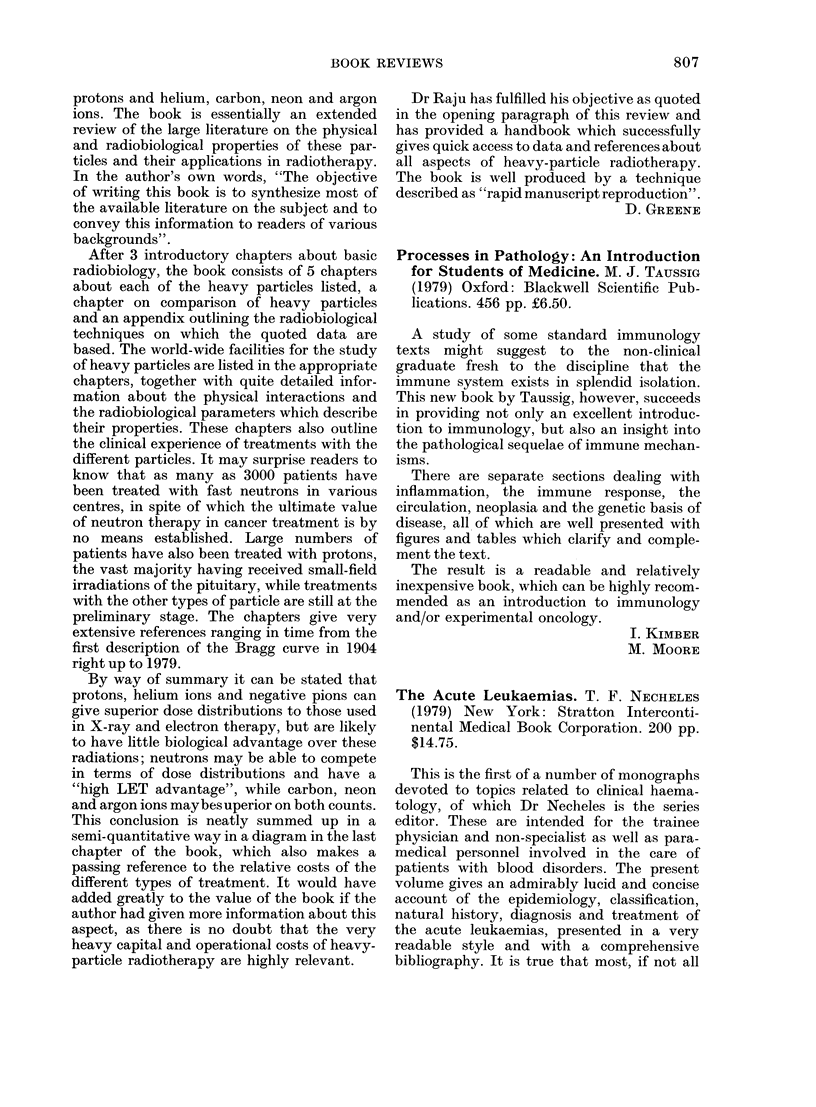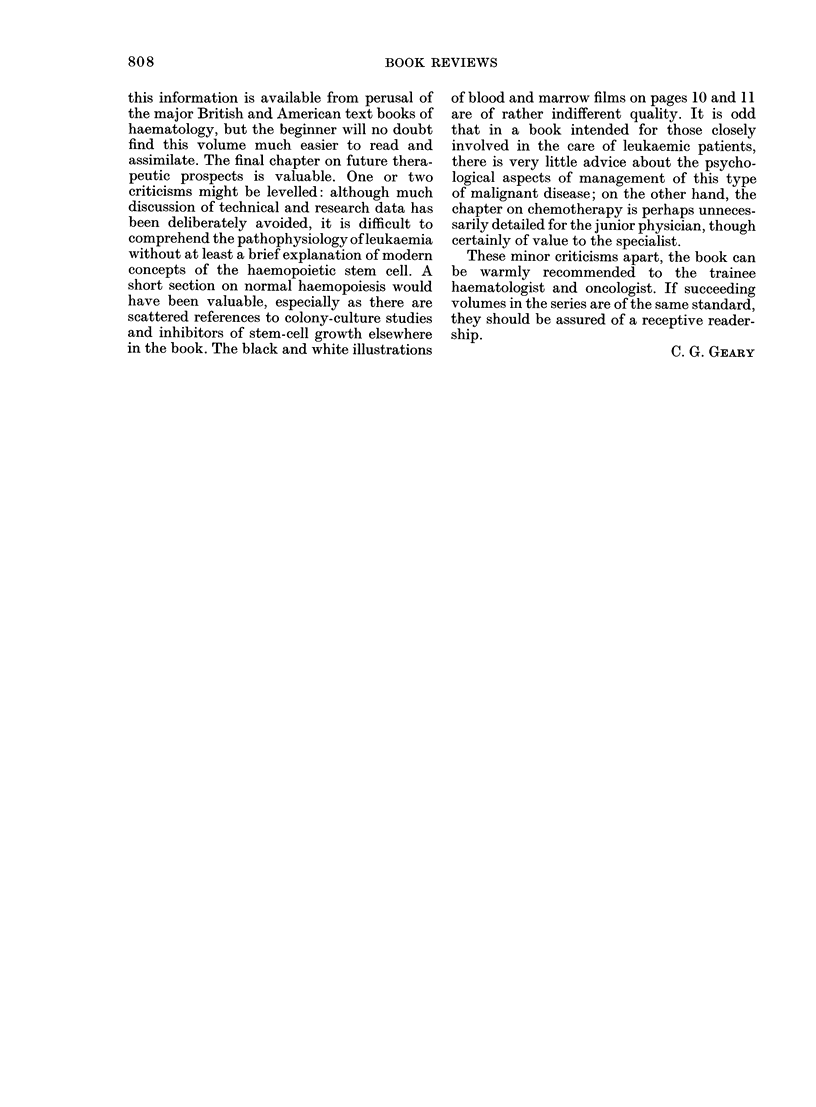# The Acute Leukaemias

**Published:** 1980-11

**Authors:** C. G. Geary


**The Acute Leukaemias.** T. F. Necheles (1979) New York: Stratton Intercontinental Medical Book Corporation. 200 pp. $14.75.

This is the first of a number of monographs devoted to topics related to clinical haematology, of which Dr Necheles is the series editor. These are intended for the trainee physician and non-specialist as well as paramedical personnel involved in the care of patients with blood disorders. The present volume gives an admirably lucid and concise account of the epidemiology, classification, natural history, diagnosis and treatment of the acute leukaemias, presented in a very readable style and with a comprehensive bibliography. It is true that most, if not all this information is available from perusal of the major British and American text books of haematology, but the beginner will no doubt find this volume much easier to read and assimilate. The final chapter on future therapeutic prospects is valuable. One or two criticisms might be levelled: although much discussion of technical and research data has been deliberately avoided, it is difficult to comprehend the pathophysiology of leukaemia without at least a brief explanation of modern concepts of the haemopoietic stem cell. A short section on normal haemopoiesis would have been valuable, especially as there are scattered references to colony-culture studies and inhibitors of stem-cell growth elsewhere in the book. The black and white illustrations of blood and marrow films on pages 10 and 11 are of rather indifferent quality. It is odd that in a book intended for those closely involved in the care of leukaemic patients, there is very little advice about the psychological aspects of management of this type of malignant disease; on the other hand, the chapter on chemotherapy is perhaps unnecessarily detailed for the junior physician, though certainly of value to the specialist.

These minor criticisms apart, the book can be warmly recommended to the trainee haematologist and oncologist. If succeeding volumes in the series are of the same standard, they should be assured of a receptive readership.

C. G. Geary